# Adiponectin Paradox in Coronary Artery Bypass Graft Patients: A Comparative Analysis of Pericardial Fluid and Plasma Levels

**DOI:** 10.7759/cureus.93766

**Published:** 2025-10-03

**Authors:** Resat Dikme

**Affiliations:** 1 Perfusion Technology/Dialysis Program, Harran University, Şanlıurfa, TUR

**Keywords:** adiponectin, coronary artery disease, epicardial adipose tissue, paracrine effect, pericardial fluid

## Abstract

Background

Adiponectin is an anti-inflammatory and cardioprotective adipokine involved in the regulation of glucose and lipid metabolism. Although it has been extensively studied in blood, data on its concentrations in pericardial fluid remain scarce.

Objectives

This study aimed to assess adiponectin levels in plasma and pericardial fluid of patients undergoing coronary artery bypass grafting (CABG) and to evaluate their clinical significance in comparison with healthy individuals.

Methodology

Pericardial fluid and plasma samples were obtained from 50 patients who underwent CABG, while plasma samples were collected from 50 healthy controls. Adiponectin levels were measured using the enzyme-linked immunosorbent assay (ELISA) method.

Results

In the patient group, the plasma adiponectin level was 7.68±1.76 ng/mL (median: 7.70), while the pericardial fluid level was 8.70±3.16 ng/mL (median: 8.56). The plasma level in the healthy control group was 5.25±0.77 ng/mL (median: 5.20), significantly lower than that in the patient group (p<0.001). No significant difference was observed between patient plasma and pericardial fluid levels (p=0.070). The receiver operating characteristic (ROC) analysis revealed a high discriminative power of plasma adiponectin levels in distinguishing patients from controls, with an area under the curve (AUC) value of 0.901.

Conclusion

Elevated adiponectin levels in both plasma and pericardial fluid of CABG patients suggest regulation by systemic and local mechanisms, possibly via epicardial adipose tissue. This pattern aligns with the “adiponectin paradox,” where increased levels may reflect a compensatory response or cardiac stress in advanced disease. Pericardial fluid assessment provides novel insights into the cardiac microenvironment with potential diagnostic and prognostic value.

## Introduction

Coronary artery disease (CAD) is among the most prevalent cardiovascular disorders worldwide and remains a leading cause of mortality and morbidity [1]. Its incidence continues to rise, not only due to population aging but also because of increasing risk factors such as obesity, diabetes, and dyslipidemia, with a notable impact even on younger adults and women [2-4]. This growing burden underscores the need to explore biomarkers and molecular mechanisms relevant to CAD pathogenesis.

Adiponectin, an adipokine predominantly secreted by adipose tissue, plays a key role in regulating energy metabolism, insulin sensitivity, and inflammation [5,6]. In cardiovascular diseases, it exerts anti-inflammatory, anti-atherogenic, and cardioprotective effects, including endothelial protection and reduction of oxidative stress [5-9]. Previous studies have therefore evaluated serum adiponectin in relation to cardiometabolic diseases and coronary heart disease, highlighting its potential as a biomarker for cardiac remodeling, arrhythmias, and other pathophysiological processes [7,10,11].

Nevertheless, the relationship between adiponectin and cardiovascular disease is complex. Low levels have been associated with myocardial infarction, atherosclerosis, hypertrophy, hypertension, and impaired ventricular function [9,12-18], whereas paradoxically high levels in CAD patients have been linked to increased cardiovascular and all-cause mortality, a phenomenon termed the “adiponectin paradox,” particularly evident in advanced heart failure [17-19]. Genetic studies further suggest that these associations may reflect secondary factors rather than a direct causal role [18-20].

More recently, a negative correlation between epicardial adipose tissue volume and serum adiponectin has been reported, underscoring its relevance for cardiovascular risk assessment [11]. Because pericardial fluid reflects the cardiac microenvironment more directly than plasma, it may provide unique insight into local inflammatory and metabolic changes [21]. Evaluating adiponectin in both plasma and pericardial fluid of coronary artery bypass grafting (CABG) patients may therefore contribute to a more comprehensive understanding of CAD pathophysiology.

## Materials and methods

Study population

This study included 50 patients scheduled for CABG and 50 healthy volunteers. The patient group consisted of individuals in stable clinical condition with an elective CABG procedure planned. Patients undergoing emergency surgery due to acute myocardial infarction or unstable angina were excluded. Additional exclusion criteria included a history of active infection, malignancy, rheumatologic disease, or advanced organ failure (e.g., end-stage renal or hepatic failure). The control group comprised healthy volunteers without any known chronic disease, with normal findings in routine health check-ups, and no prior diagnosis of cardiovascular disease. Blood and pericardial fluid samples were collected from the patient group, whereas only blood samples were collected from the control group.

Blood sampling procedure

In the patient group, venous blood samples were obtained before CABG surgery, and in the control group, samples were collected at the time of recruitment. Samples were transferred into ethylenediaminetetraacetic acid (EDTA) tubes, centrifuged at 5000 rpm for five minutes, and plasma was separated. Plasma samples were stored at -80°C until analysis.

Pericardial fluid sampling procedure

Pericardial fluid samples were collected during CABG surgery according to the standard open-heart surgery protocol. Following sternotomy and pericardial incision, accumulated pericardial fluid was aspirated under sterile conditions before any irrigation was performed. Approximately 3-10 mL of pericardial fluid was aspirated using a sterile syringe, transported to the laboratory at 4°C, and placed in sterile, EDTA-free tubes to prevent contamination. Samples were centrifuged at 3000 rpm for five minutes to remove cellular components, and the supernatant was transferred to another sterile tube and stored at -80°C until analysis.

Laboratory analyses

Adiponectin concentrations in pericardial fluid and plasma were quantitatively determined using a human adiponectin enzyme-linked immunosorbent assay (ELISA) kit (FineTest (Wuhan Fine Biotech Co., Ltd., Wuhan, China), Catalogue No.: EH2593; range: 1.563-100 ng/mL; sensitivity: 0.938 ng/mL) in accordance with the manufacturer’s instructions. All measurements were performed in duplicate, and the mean values were used for analysis.

Statistical analysis

All statistical analyses were conducted using IBM SPSS Statistics software, version 25.0 (IBM Corp., Armonk, NY, USA). The distribution of continuous variables was assessed with the Shapiro-Wilk test. As adiponectin levels did not follow a normal distribution, non-parametric tests were applied for group comparisons. The Kruskal-Wallis test was used to evaluate overall differences among groups, and pairwise comparisons were performed using the Mann-Whitney U test with Bonferroni correction for multiple testing. Effect sizes for pairwise comparisons were calculated using rank-biserial correlation (r). Diagnostic accuracy of adiponectin levels was evaluated by receiver operating characteristic (ROC) curve analysis, and the area under the curve (AUC) was calculated. A p-value < 0.05 was considered statistically significant.

## Results

The demographic and clinical characteristics of the patient and control groups are presented in Table 1. There were no statistically significant differences between groups regarding age, height, weight, or body surface area (p>0.05 for all). The gender distribution was comparable, with 46% females and 54% males in the patient group and 40% females and 60% males in the control group. In the patient group, 68% had at least one comorbidity (diabetes mellitus, hypertension, or both), whereas all participants in the control group were free of chronic diseases.

**Table 1:** The demographic and clinical characteristics of the patient and control groups BSA: body surface area; Continuous variables are presented as mean ± SD; categorical variables as n (%). “–” indicates not applicable. The n is the number of patients. A p-value of <0.05 was considered statistically significant.

Variable	Patient Group (n=50)	Control Group (n=50)	p-value
Age (years), mean ± SD	60.23 ± 6.43	57.89 ± 6.21	0.0657
Height (cm), mean ± SD	168.37 ± 8.45	170.22 ± 8.06	0.0562
Weight (kg), mean ± SD	78.96 ± 11.87	79.42 ± 12.76	0.0733
BSA (m²), mean ± SD	1.91 ± 0.21	1.93 ± 0.19	0.0742
Female, n (%)	23 (46.0%)	20 (40.0%)	0.0634
Male, n (%)	27 (54.0%)	30 (60.0%)	0.0589
Comorbidities, n (%)			
Diabetes mellitus	17 (34.0%)	–	–
Hypertension	9 (18.0%)	–	–
Diabetes + hypertension	8 (16.0%)	–	–
No comorbidities	16 (32.0%)	50 (100.0%)	<0.001

Of 50 patients in the patient group, there were 23 females (46.0%) and 27 males (54.0%). The control group included 20 females (40.0%) and 30 males (60.0%) patients. Regarding comorbidities, 17 (34.0%) had diabetes mellitus, nine (18.0%) had hypertension, eight (16.0%) had both, and 16 (32.0%) had no comorbidities. All controls were free of chronic disease (50/50, 100.0%).

Descriptive statistics of adiponectin levels for the patient and control groups are presented in Table 2. Adiponectin concentrations were highest in the pericardial fluid of patients, followed by patient plasma, and lowest in the plasma of healthy controls.

**Table 2:** Adiponectin levels in patient and control groups Values are presented as mean ± SD and median (min–max). min: minimum; max: maximum

Group	Adiponectin (ng/mL) Mean ± SD	Adiponectin (ng/mL) Median (Min–Max)
Patient plasma	7.68±1.76	7.70 (2.46–13.17)
Patient pericardial fluid	8.70±3.16	8.56 (2.30–18.83)
Control plasma	5.25±0.77	5.20 (2.05–7.67)

Pericardial fluid samples from patients showed the highest adiponectin levels in both mean (8.70±3.16 ng/mL) and median (8.56 ng/mL) values. Patient plasma levels were slightly lower (7.68±1.76 ng/mL; median: 7.70 ng/mL) but remained markedly higher than those of controls. Plasma from healthy controls exhibited the lowest mean (5.25±0.77 ng/mL) and median (5.20 ng/mL) adiponectin concentrations.

The distribution of adiponectin levels in each group was assessed using the Shapiro-Wilk test, which indicated that none of the groups followed a normal distribution (p<0.05). Therefore, the non-parametric Kruskal-Wallis test was applied for group comparisons, revealing a statistically significant difference among the groups (H= 65.367 and degrees of freedom = 2, (p<0.001)). This finding suggests that at least one group differed from the others. To determine which groups differed, post-hoc pairwise comparisons were performed using the Mann-Whitney U test, with Bonferroni correction applied to reduce the risk of Type I error due to multiple testing. The results are summarized in Table 3.

**Table 3:** Pairwise comparisons of adiponectin levels between groups using the Mann–Whitney U test Pairwise comparisons of adiponectin levels between groups performed using the Mann–Whitney U test with Bonferroni correction. A p-value of <0.05 was considered statistically significant.

Comparison	p-value
Patient plasma vs. Patient pericardial fluid	0.070
Patient plasma vs. Control plasma	<0.001

Pairwise analysis showed no statistically significant difference between patient plasma and pericardial fluid adiponectin levels, despite the higher median values in the pericardial fluid (p=0.070). In contrast, plasma adiponectin levels in patients were significantly higher than those in healthy controls (p<0.001), indicating elevated circulating adiponectin concentrations in CAD compared with healthy individuals.

Following the Mann-Whitney U tests, effect size analysis was performed to assess the magnitude of differences between groups. Rank-biserial correlation coefficients (r) were calculated for each pairwise comparison, and the results are presented in Table 4.

**Table 4:** Effect sizes (Rank-biserial correlation) based on Mann–Whitney U tests *p<0.05, statistically significant. Effect sizes (rank-biserial correlation, r) for pairwise group comparisons. Interpretation: small (0.10–0.29), medium (0.30–0.49), large (≥0.50).

Comparison	Effect Size (r)
Patient plasma vs. Pericardial fluid	–0.181
Patient plasma vs. Control plasma	0.695*

The comparison between patient plasma and control plasma showed a large effect size (r = 0.695), indicating not only statistical significance but also clinical relevance. In contrast, the effect size between patient plasma and pericardial fluid was small (r=-0.181) and not statistically significant.

ROC analysis and diagnostic performance

**Figure 1:**
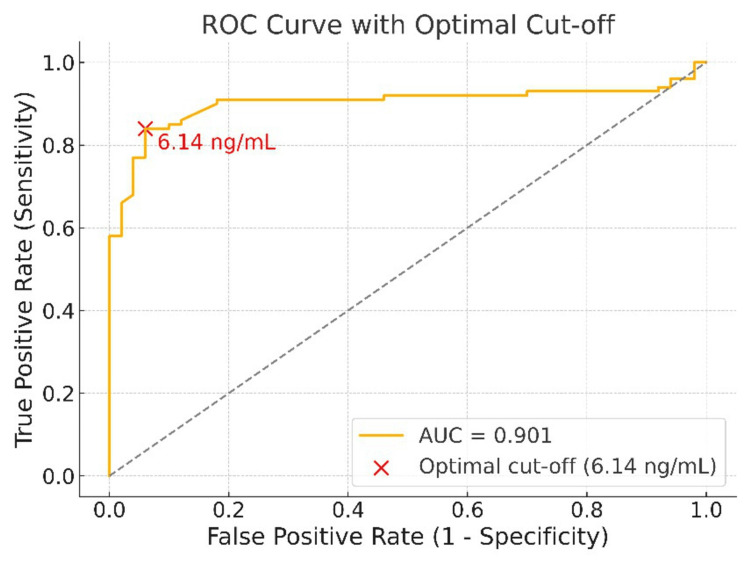
ROC curve of adiponectin levels for distinguishing CABG patients from healthy controls (AUC = 0.901) Receiver operating characteristic (ROC) curve of plasma adiponectin levels for distinguishing coronary artery bypass grafting (CABG) patients from healthy controls. The area under the curve (AUC) was 0.901. The optimal cut-off identified by the Youden index was 6.14 ng/mL, yielding 84% sensitivity and 94% specificity.

An ROC analysis was performed to evaluate the ability of adiponectin levels to distinguish between the patient and healthy control groups. The AUC was calculated as 0.901, indicating a high level of diagnostic discrimination (Figure 1).

According to the ROC analysis, the optimal cut-off value determined by the Youden index was 6.14 ng/mL. At this threshold, sensitivity and specificity were calculated as 84% and 94%, respectively. These findings suggest that adiponectin not only shows a statistically significant difference between groups but also holds strong potential as a diagnostic biomarker for the disease.

## Discussion

Adiponectin exists in several isoforms, namely, high- (HMW), medium- (MMV), low-molecular-weight (LMW), and globular, each with distinct biological activities [22]. The HMW form is particularly insulin-sensitizing and anti-atherogenic, whereas other isoforms show more limited effects. Isoform-specific targeting has therefore been proposed as a potential therapeutic strategy.

Low adiponectin levels are linked to cardiovascular complications in obesity, insulin resistance, and diabetes [23]. Conversely, elevated adiponectin has been associated with adverse outcomes, including ischemic stroke [24] and poorer prognosis in CAD and heart failure cohorts; this pattern has been termed the “adiponectin paradox” [25, 26]. These elevations may reflect a compensatory response or a marker of disease severity.

Some studies also suggest protective effects in certain populations (e.g., older adults or specific genetic backgrounds), where higher adiponectin may be associated with reduced myocardial infarction risk [27]. Overall, adiponectin dynamics should be interpreted in light of comorbidities and disease stage.

Similarities and differences of findings with the literature

In our study, adiponectin levels in both plasma and pericardial fluid were higher in patients than in healthy controls, consistent with reports linking elevated adiponectin in CAD to worse outcomes and advanced disease [24,26]. These findings may therefore represent another example of the “adiponectin paradox.”

However, this contrasts with earlier evidence suggesting that adiponectin is typically reduced in atherosclerotic disease, particularly with obesity or type 2 diabetes [23]. Data on pericardial fluid remain scarce. Elie et al. observed lower pericardial fluid adiponectin than plasma in mixed cardiac surgery patients [28], whereas our CABG-only cohort showed higher pericardial levels. This discrepancy may reflect differences in patient selection or methodology.

Although epicardial adipose tissue in CAD is known to downregulate adiponectin and upregulate inflammatory cytokines [29], protein concentrations in pericardial fluid may not directly mirror gene expression. Post-transcriptional and translational regulation, protein degradation, and secretion dynamics can all produce divergence between mRNA expression and protein levels [30,31]. Thus, the elevated pericardial adiponectin observed in our study may reflect such regulatory mechanisms and underscores the need for further research to clarify its origin and role.

Possible reasons for elevated adiponectin in the patient group

Adiponectin in pericardial fluid may partly reflect diffusion from plasma, where levels were already elevated, but could also arise from local secretion by epicardial and perivascular adipose tissue. Because patients with metabolic comorbidities such as obesity or chronic kidney disease, conditions known to suppress adiponectin, were excluded, other factors such as a distinct metabolic milieu or genetic/environmental influences may have contributed to the increase [28].

In advanced cardiac disease, a compensatory anti-inflammatory response may also enhance adiponectin production to counteract CAD-related stress [32,33]. Such elevations, however, have paradoxically been linked to poor prognosis, consistent with the “adiponectin paradox.” Overall, these mechanisms suggest that adiponectin dynamics depend not only on fat mass but also on disease severity and the body’s adaptive capacity.

Importance of measuring adiponectin in pericardial fluid

Pericardial fluid, in direct contact with the heart, reflects the cardiac microenvironment and contains hormones, cytokines, and growth factors [34]. In CAD and heart failure, some biomarkers reach higher concentrations here than in plasma [28], making their analysis valuable for understanding local pathophysiology.

In this study, assessing adiponectin in pericardial fluid provides a novel perspective beyond plasma measurements. Elevated levels may reflect local mechanisms or disease phenotypes, offering insights independent of systemic circulation and underscoring the potential diagnostic and prognostic relevance of adiponectin in cardiovascular disease.

Limitations of the study

This study has several limitations. First, the relatively small sample size limits the generalizability of the findings, and validation in larger, multicenter studies is required. Second, inclusion was restricted to patients undergoing CABG surgery, which limits the applicability of the results to other cardiovascular patient groups. Third, metabolic markers that may influence adiponectin levels, such as insulin resistance, visceral fat volume, genetic variations, and other adipokines, were not assessed. In the control group, cardiometabolic risk parameters such as body mass index, lipid profile, and insulin sensitivity were not measured, making it difficult to determine whether the observed differences were due solely to disease status or to underlying risk factors.

Additionally, the ELISA kit used measured total adiponectin without distinguishing between isoforms, which may limit comparisons with literature focusing on the HMW isoform. The kit’s measurement range (1.563-100 ng/mL) and sensitivity (0.938 ng/mL) may affect accuracy at very low or high concentrations. Finally, only adiponectin levels were evaluated; functional and cellular-level analyses are needed to better understand the cardiovascular effects of this molecule.

## Conclusions

This study demonstrates that adiponectin levels are elevated in both plasma and pericardial fluid of CABG patients compared with healthy controls, consistent with the adiponectin paradox. These elevations may reflect systemic and local regulatory mechanisms, possibly representing a compensatory response in advanced disease. Pericardial fluid analysis offers additional insight into the cardiac microenvironment, although its diagnostic and prognostic value remains to be clarified. Further multicenter studies are needed to validate these findings and determine their clinical relevance.
